# Dexmedetomidine decreased the post‐thyroidectomy bleeding by reducing cough and emergence agitation – a randomized, double‐blind, controlled study

**DOI:** 10.1186/s12871-021-01325-6

**Published:** 2021-04-12

**Authors:** Sang Hun Kim, Yoo Seok Kim, Seongcheol Kim, Ki Tae Jung

**Affiliations:** 1 Department of Anesthesiology and Pain Medicine, Chosun University Hospital, 365 Pilmun-dearo, Donggu, 61453 Gwangju, Korea; 2 Department of Anesthesiology and Pain Medicine, College of Medicine and Medical School, Chosun University, Gwangju, Korea; 3 Department of Surgery, Chosun University College of Medicine, Chosun University Hospital, Gwangju, Korea

**Keywords:** Cough, Dexmedetomidine, Hemorrhage, Ramsey sedation scale, Recovery, Thyroidectomy

## Abstract

**Background:**

Bleeding after thyroidectomy occurs due to violent coughing during emergence. Dexmedetomidine is helpful for the smooth emergence and suppression of cough. The purpose of the present study was to compare the effects of dexmedetomidine on postoperative bleeding after thyroidectomy.

**Methods:**

Randomized, double-blind, controlled trials were conducted in female patients (ASA I–II, aged 20 to 60 years). The patients were randomly allocated into two groups. Approximately 15 min before the end of the surgery, dexmedetomidine was administered (0.6 µg/kg/h) without a loading dose in group D (*n* = 69), and normal saline was administered in group S (*n* = 70) at the same infusion rate. Hemodynamic data, coughing reflex, extubation time, Ramsay sedation scale (RSS), and recovery time were assessed during the administration of the study drugs and recovery from anesthesia. The amount of postoperative hemorrhage was measured for 3 days.

**Results:**

Data from a total of 139 patients were analyzed. The incidence of severe cough was significantly lower in group D than in group S (4.3 % vs. 11.5 %, *P* = 0.022). The emergence agitation in the postanesthetic care unit was significantly lower in group D than in group S (*P* = 0.01). Postoperative bleeding was significantly lower in group D than in group S until the second postoperative day (*P* = 0.015).

**Conclusions:**

Dexmedetomidine can be helpful in decreasing bleeding after thyroidectomy by reducing coughing and emergence agitation.

**Trial registration:**

This study was registered at http://clinicaltrials.gov (registration number NCT02412150, 09/04/2015).

## Background

Although the occurrence of bleeding after thyroidectomy is relatively low (0–4.2 %), it is regarded as a severe complication that can be life-threatening and requires immediate treatment [1]. Many cases of post-thyroidectomy bleeding occur due to violent cough that develops particularly while waking up from anesthesia and during extubation [2]. Various trials have examined the effects of administering remifentanil or dexmedetomidine on suppressing cough during the extubation period and emergence [3, 4].

Dexmedetomidine, a highly selective α_2_-receptor agonist, has recently gained attention as an intraoperative adjuvant in various situations. Dexmedetomidine helps decrease emergence agitation and helps keep a patient in a calm state after surgery [5, 6]. Moreover, a small dose of dexmedetomidine is effective in suppressing cough during emergence from anesthesia without respiratory depression [4]. Thus, a small dose of dexmedetomidine may also reduce postoperative bleeding after thyroidectomy by reducing cough and emergence agitation. However, no studies have shown if the effect of dexmedetomidine on reducing emergence agitation and cough can reduce postoperative bleeding after thyroidectomy.

The purpose of the present study was to determine the effect of dexmedetomidine, administered during emergence without a loading dose in female patients undergoing elective thyroidectomy, on postoperative bleeding by reducing coughing and emergence agitation.

## Methods

This randomized, double-blind, controlled study was conducted after it was approved by the Institutional Review Board of Chosun University Hospital (2014-04-004) and was registered at http://clinicaltrials.gov (registration number NCT02412150, 09/04/2015).

A total of 139 female patients who were undergoing elective total thyroidectomy under general anesthesia (ASA class 1–2, aged over 20–60 years) in our hospital were enrolled in the study. Patients with the following conditions were excluded: risk of a difficult airway, history of respiratory disease, chronic cough, cardiovascular disease, or pregnant or breast-feeding woman. All patients agreed to participate in the study after careful explanation, and written informed consent for participation in the study was obtained. The recruited patients were randomly allocated in a 1:1 ratio according to computer-generated random numbers, and sealed envelopes were prepared by an independent anesthesiologist. When the patients agreed to participate in the study, the envelopes were opened in sequential order and the patients were allocated according to the number into two groups: Group D (*n* = 69): Dexmedetomidine (Precedex®^;^ Pfizer, New York, NY, USA) was administered (0.6 µg/kg/h) after stopping the administration of remifentanil 15 min before the end of surgery; Group S (*n* = 70): Normal saline was administered as a control in the same way. For blindness, an independent nurse and anesthesiologist who did not participate in the anesthetic procedure prepared the study drugs and assessed the outcomes. Dexmedetomidine, which was diluted to a 50 mL volume (diluted to 0.2 µg/mL) and normal saline were prepared in a code-labeled 50-mL syringe according to the coded number of the patients.

Patients were advised to fast overnight and were administered intramuscular midazolam (0.05 mg/kg) before being transferred to the operating room (OR). When the patients arrived at the OR, a monitoring device (Carescape; GE Healthcare, USA) was used to perform electrocardiograms, measure blood pressure in a non-invasive way, and perform pulse oximetry and neuromuscular and bispectral index (BIS) monitoring. The induction of anesthesia was performed by a skilled anesthesiologist who was blinded to the allocation of the patient. For the induction, 2.0 mg/kg propofol was administered and a targeted effect-site concentration (Ce) of remifentanil was adjusted as 2.0 ng/mL using a target-controlled infusion device (Orchestra® Base Primea; Fresenius-Vial, France) based on a Minto pharmacokinetic model. When the patients lost their consciousness, rocuronium bromide (0.8 mg/kg) was administered and endotracheal intubation with an armored tube (internal diameter: 7.0 mm) was performed after confirming adequate neuromuscular blockade by a train-of-four (TOF) ratio of 0 and no neuromuscular blocker was used during the surgery. To maintain anesthesia, desflurane with a 50 % O_2_-air mixture was used, and the end-tidal concentration of desflurane and the Ce of remifentanil were adjusted according to the BIS score (between 40 and 60) and vital signs (within 20 % of baseline values). The initial tidal volume was set at 8 mL/kg with respiratory rates of 12 breaths per min, which were adjusted to maintain the end-tidal CO_2_ between 35 mmHg and 40 mmHg and peak inspiratory pressure below 28 mmHg.

When the surgeon performed the subcutaneous suture, which was approximately 15 min before the end of the surgery, the infusion of remifentanil was discontinued, and a code-labeled syringe was prepared and infused at a rate of 3 mL/kg/h until the patient was fully awake and transferred to the post-anesthetic care unit (PACU). When the surgeon ended the suture, desflurane was discontinued approximately 5 min before the end of the surgery and the patient was ventilated with 100 % O_2_ (5 L/min). To control postoperative pain, fentanyl was administered with a patient-controlled analgesia instrument according to the hospital protocol (basal infusion, 0.625 µg/kg/h without a loading dose; intermittent bolus, 1.0 µg/kg/h; lockout time, 15 min). After the use of reversal agents [pyridostigmine (0.15 mg/kg) with glycopyrrolate (0.2 mg/5 mg of pyridostigmine)], recovery from neuromuscular blockade was confirmed using a neuromuscular monitor (TOF ratio > 99 %). During recovery, the patients were asked to open their eyes by verbal request without any other stimulation or disturbance. When the patient regained spontaneous ventilation and consciousness (BIS score > 90), careful extubation was performed while avoiding irritation, and the patient was transferred to the PACU.

The primary objective of the study was to measure the amount of postoperative bleeding for three consecutive days. The amount of postoperative blood that was collected in the drainage system was measured by an independent nurse before leaving the PACU and measured at the ward at 24 h intervals. The amount of blood in each period was measured after emptying the blood collected from the previous period in the drainage. Secondary outcomes such as vital signs, extubation time, recovery time, cough reflex, Ramsay Sedation Scale (RSS), 11-point numeric rating scale (NRS, 0 = no pain and 10 = worst pain imaginable) for pain measurement, etc., were assessed by independent anesthesiologists and surgeons. The patient characteristics, duration of surgery, duration of infusion of study drugs, and amount of fluid administered during the surgery were recorded. Vital signs such as mean blood pressure (MBP) and heart rate (HR) were measured according to the time interval as follows: T0, before the administration of the study drugs; T1, 5 min after the administration of the study drugs; T2, 10 min after the administration of the study drugs; T3, 15 min after the administration of the study drugs; T4, just before extubation; T5, 5 min after extubation; T6, after arrival at the PACU. During recovery from anesthesia (time interval from discontinuing desflurane to transfer to the PACU), the cough reflex was measured visually and graded according to the severity (grade 0, no cough; grade 1, single cough with mild severity; grade 2, cough persistence less than 5 s with moderate severity; grade 3, severe, persistent cough for more than 5 min) [7]. Extubation time (time interval from the discontinuation of desflurane to extubation) and recovery time (time interval from the discontinuation of desflurane to transfer to the ward) were assessed. Approximately 5 min after arriving at the PACU, the RSS of the patient was measured as follows: 1, patient anxious and agitated or restless or both; 2, patient cooperative, oriented, and tranquil; 3, the patient responds to commands only; 4, asleep or a brisk response to a light glabellar tap or loud auditory stimulus; 5, sluggish response to a light glabellar tap or loud auditory stimulus; 6, no response to a light glabellar tap or loud auditory stimulus [8]. The patients were also classified according to the RSS as follows: agitated, RSS 1; calm, RSS 2–3; sedated, RSS 4–6 [9]. In the PACU, the incidence of desaturation (< 90 %) was assessed as an adverse effect of dexmedetomidine. After the patient was transferred to the ward, the amount of postoperative bleeding and pain score using NRS were assessed daily until the third postoperative day (POD). The duration of drainage catheter placement after surgery was also recorded.

The sample size was calculated using “G*Power3” free software. The effect size was calculated based on a previous study in which the incidence of cough was 55 % after a single use of dexmedetomidine infusion [4]. The total sample size was calculated to be 136 with a calculated effect size of 0.441, α = 0.05, and a power of 80 %. The drop-out rate was assumed to be 10 %, and 70 patients were allocated to each group.

Statistical analyses were performed using IBM SPSS Statistics for Windows, version 21.0 (IBM Corp., Armonk, NY, USA). Normality was assessed using the Kolmogorov-Smirnov test and the Shapiro-Wilk test. Values are expressed as the mean (SD), median (interquartile range), or number of patients (%) with exact P values. Normally distributed data (age, height, weight, and BMI) were analyzed using Student’s *t*-test. Non-normally distributed data (duration of surgery, amount of intraoperative fluid, infusion duration of study drug, extubation time, recovery time, and duration of the drainage catheter placement) were analyzed using the Mann–Whitney U-test. Categorical variables (ASA class, coexisting disease, grades of cough response, incidence of severe cough, and RSS) were analyzed using either the Chi-squared or Fisher’s exact test. The change in vital signs, NRS score, and amount of postoperative bleeding according to the time sequence were analyzed by a repeated-measures two-way ANOVA, and post-hoc testing was performed using the Mann–Whitney U-test. The odds ratio, relative risk, and risk differences with 95 % confidence intervals (95 % CI) were calculated as a measure to compare the risk of severe cough and agitated state in the PACU according to the RSS associated with the use of dexmedetomidine. Differences were considered statistically significant when the P value was less than 0.05.

## Results

**Fig. 1:**
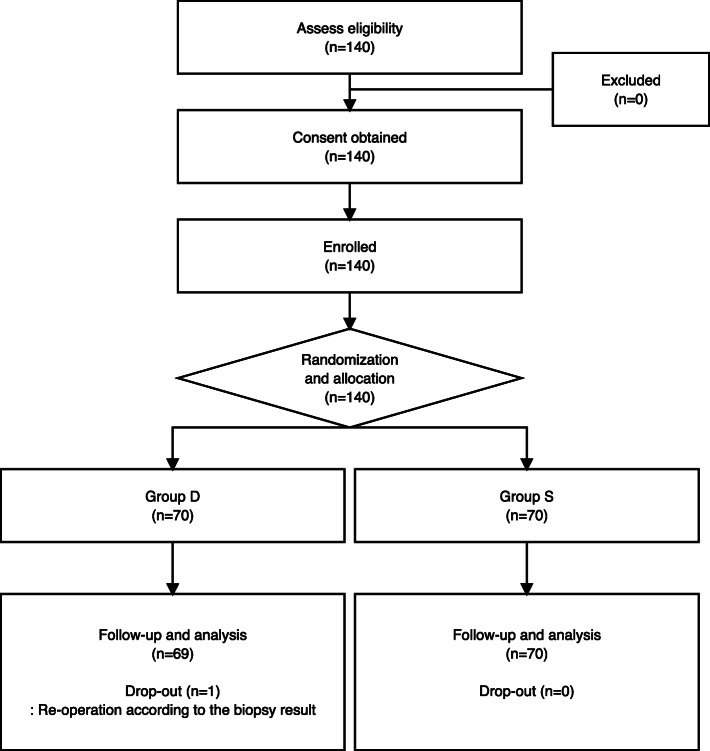
CONSORT flow diagram for the study. Group D was administered dexmedetomidine (0.6 µg/kg/h); Group S was administered normal saline as a control

A total of 140 female patients scheduled for elective thyroidectomy were assessed for eligibility. Among the 140 patients, none did not meet the inclusion criteria or refused to participate. A total of 140 patients were enrolled, but one patient in group D was excluded because of re-operation according to the biopsy results. Finally, data from 139 patients (group D, *n* = 69; group S, *n* = 70) were analyzed (Fig. 1).

**Fig. 2:**
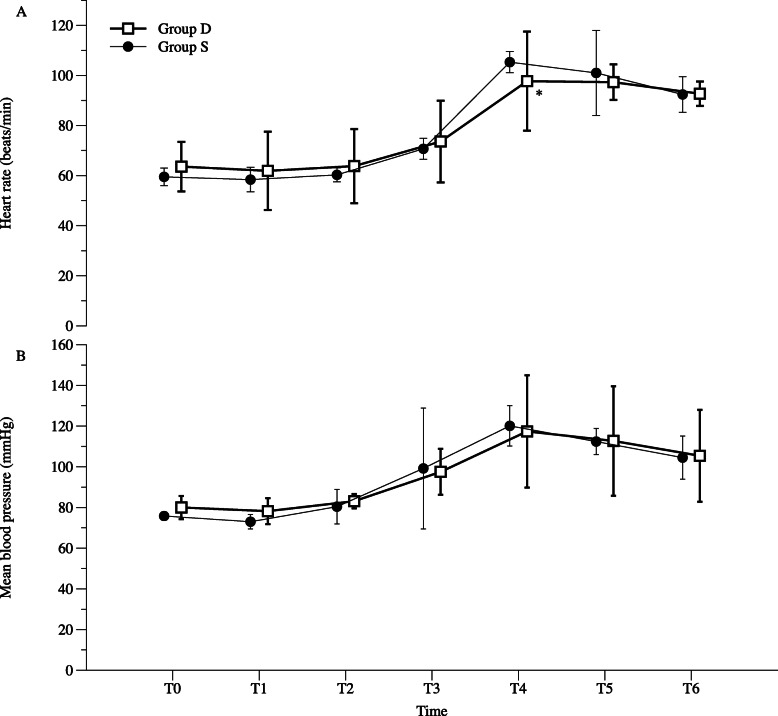
Hemodynamic changes during the administration of the study drugs and emergence from anesthesia. **a** mean blood pressure and **b** heart rate measured. T0, before the administration of the study drugs; T1, 5 min after the administration of the study drugs; T2, 10 min after the administration of the study drugs; T3, 15 min after the administration of the study drugs; T4, just before extubation; T5, 5 min after extubation; T6, after arrival at the postanesthetic care unit. Group D was administered dexmedetomidine (0.6 µg/kg/h); Group S was administered normal saline as a control. *P < 0.05 compared with group S

There were no significant differences in patient characteristics, duration of surgery, amount of intraoperative fluid, and infusion duration of study drugs between the two groups (Table 1). The MBP and HR of both groups showed little change during the infusion of the study drugs, which increased during the periods of extubation (Fig. 2). There were no significant differences in MBP between the two groups (*P* = 0.143). The HR was significantly different between the two groups (*P* = 0.001). Just before extubation, the HR of group D was significantly lower than that of group S (*P* = 0.015, Fig. 2a).

**Table 1:** Patient characteristics and intraoperative variables

	Group D (*n* = 69)	Group S (*n* = 70)	*P*-value
Age (yr)	44.2 (1.4)	45.0 (4.9)	0.646
Height (cm)	158.8 (5.7)	159.5 (1.4)	0.444
Weight (kg)	61.7 (7.8)	61.4 (6.4)	0.909
BMI	24.1 (5.2)	23.8 (2.1)	0.652
ASA class (I/II)	49/20	51/19	0.852
Coexisting disease			
Hypertension	9 (13.0)	11 (15.7)	0.810
Diabetes	3 (4.3)	4 (5.7)	1.000
Renal disease	0 (0)	1 (1.4)	1.000
Duration of surgery (min)	115.0 [45.0]	115.0 [35.0]	0.947
Amount of intraoperative fluid (mL)	300.0 [100.0]	300.0 [50.0]	0.779
Infusion duration of study drug (min)	34.0 [8.0]	32.0 [22.0]	0.539

Values are expressed as mean (standard deviation), median (interquartile range), or number (%). Group D was administered dexmedetomidine (0.6 µg/kg/h); Group S was administered normal saline as a control

*BMI* body mass index, *ASA* American Society of Anesthesiologists

The emergence profiles are presented in Table 2. There were no significant differences in extubation time (*P* = 0.728) and recovery time (*P* = 0.604). The cough reflex was significantly different between the two groups (*P* = 0.015), and the incidence of severe cough (grade 3) was significantly lower in group D than in group S (*P* = 0.022). The odds ratio of severe cough was 0.321 (95 % CI, 0.118–0.879) in group D. The relative risk of severe cough was 0.635 (95 % CI, 0.460–0.876) in group D and 1.974 (95 % CI, 0.978–3.987) in group S. The risk difference between the two groups was 1.339 (95 % CI, 1.277–1.401). The RSS also showed significant differences between the groups (*P* < 0.022). According to the RSS classification, the patients in group D maintained a calmer state (36.0 % in group D vs. 29.5 % in group S, *P* = 0.01) in the PACU. In particular, the patients in group D showed a lower incidence of the agitated state compared to the control in the PACU (7.9 % in group D vs. 20.1 % in group S). The odds ratio of the agitated state was 0.284 (95 % CI, 0.127–0.635) for group D. The relative risk of the agitated state was 0.585 (95 % CI, 0.432–0.792) in group D and 2.056 (95 % CI, 1.409–1.533) in group S. However, the risk difference between the two groups was 1.471 (95 % CI, 1.409–1.533). There was no event of oxygen desaturation in the PACU in either group.

**Table 2:** Emergence profile during awake and in the postanesthetic care unit

	Group D (*n* = 69)	Group S (*n* = 70)	*P*-value
Extubation time (min)	10.0 [8.0]	8.0 [5.25]	0.728
Recovery time (min)	41.0 [16.0]	42.0 [13.0]	0.604
Cough reflex (grade 0/1/2/3)	21/29/13/6	12/20/22/16	0.015
Incidence of severe cough (grade 3)	6 (4.3)	16 (11.5)	0.022
Odds ratio (95% CI)	0.321 (0.118, 0.879)	Referent	
Relative risk (95% CI)	0.635 (0.460, 0.876)	1.974 (0.978, 3.987)	
Risk difference (95% CI)	Referent	1.339 (1.277, 1.401)	
RSS at PACU	2.3 (0.7)	1.7 (1.4)	0.002
Classified RSS			0.01
Agitated (RSS 1)	11 (7.9)	28 (20.1)	
Calm (RSS 2–3)	50 (36.0)	41 (29.5)	
Sedated (RSS 4–7)	8 (5.8)	1 (0.7)	
Agitated RSS (RSS 1)	11 (7.9)	28 (20.1)	
Odds ratio (95% CI)	0.284 (0.127, 0.635)	Referent	
Relative risk (95% CI)	0.585. (0.432, 0.792)	2.056 (1.213, 3.486)	
Risk difference (95% CI)	Referent	1.471 (1.409, 1.533)	
Desaturation	0 (0)	0 (0)	–

Values are the mean (standard deviation), median [interquartile range], or number (%). Group D, administered dexmedetomidine (0.6 μg/kg/hr); Group S, administered normal saline as a control. Extubation time, the time interval from discontinuing the desflurane to extubate; recovery time, time interval from discontinuing the desflurane to transferred to the ward

*CI* confidence interval, *RSS* Ramsay Sedation Scale, *PACU* postanesthetic care unit. Patients were classified as agitated, RSS 1; Calm, RSS 2–3; Sedated, RSS 4–6

**Fig. 3:**
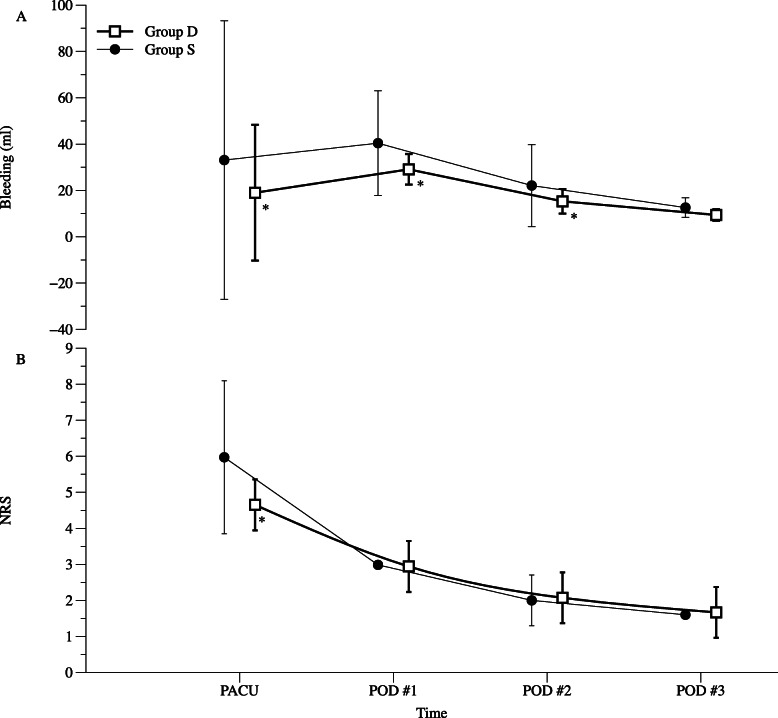
The amount of postoperative bleeding and pain score according to the time. The amount of postoperative bleeding was measured by the collected blood in the drainage system. The postoperative pain score was assessed using a numeric rating scale (NRS). PACU, postanesthetic care unit; POD, postoperative day. Group D was administered dexmedetomidine (0.6 µg/kg/h); Group S was administered normal saline as a control. *P < 0.05 compared with group S

The amount of postoperative bleeding was significantly different between the two groups (*P* = 0.015, Table 3; Fig. 3a). The amount of drained blood during emergence and duration of stay in the PACU was significantly decreased in group D compared to group S (19.0 mL vs. 33.1 mL, *P* = 0.001), and the decrease in postoperative bleeding in group D lasted for the first and second POD (*P* = 0.016 and 0.003, respectively). However, there were no significant differences in the duration of drainage catheter placement between the groups (group D: 3.7 days vs. group S: 4.0 days, *P* = 0.103).

**Table 3:** Postoperative bleeding and pain score

	Group D (*n* = 69)	Group S (*n* = 70)	*P*-value
Postoperative bleeding			0.015
PACU	19.0 (29.3)	33.1 (60.1)	0.001
POD #1	29.1 (6.6)	40.4 (22.6)	0.015
POD #2	15.3 (5.3)	22.1 (17.7)	0.003
POD #3	9.4 (0.7)	12.6 (4.2)	0.061
NRS			< 0.001
PACU	4.7 (0.7)	6.0 (2.1)	< 0.001
POD #1	2.9 (0.7)	3.0 (0.0)	0.829
POD #2	2.1 (0.7)	2.0 (0.7)	0.637
POD #3	1.7 (0.7)	1.6 (0.0)	0.420

Values are presented as means (standard deviation). Group D was administered dexmedetomidine (0.6 µg/kg/h); Group S was administered normal saline as a control

*PACU* postanesthetic care unit, *POD* postoperative day, *NRS* numeric rating scale for postoperative pain

The NRS pain score was also significantly different between the two groups (*P* < 0.001, Table 3; Fig. 3b). The NRS was significantly lower in group D than in group S at the PACU (*P* < 0.001), but there were no significant differences during POD.

## Discussion

In this study, dexmedetomidine infusion during emergence from anesthesia significantly decreased the incidence of severe cough, emergence agitation in the PACU, and the amount of bleeding that was measured by the drainage system. To our knowledge, the current study is the first to report that the administration of dexmedetomidine (0.6 µg/kg/h) without a loading dose during recovery from anesthesia is significantly associated with the decrease of postoperative bleeding after thyroidectomy.

As the thyroid gland is an organ with high blood flow, severe bleeding after thyroidectomy is related to a major life-threatening complication that requires intensive care, although the incidence of significant bleeding after thyroidectomy is as low as 2.0 % [1, 2]. In particular, hematoma formation due to bleeding after thyroidectomy can be fatal due to airway obstruction; therefore, bleeding after thyroid surgery should be observed. Even though there are numerous risk factors associated with postoperative bleeding after thyroidectomy, such as male sex, older age, and postoperative hypertension, bleeding frequently occurs with sudden violent cough during extubation and emergence [2]. Cough after thyroidectomy lifts the thyroid cartilage and loosens the ligation, leading to bleeding [2]. Even in the absence of rapid bleeding from ligated vessels, severe cough while the patient awakens can increase venous pressure to encourage bleeding from the cauterized vessels and create hematoma [10]. Moreover, thyroidectomy is associated with postoperative cough, especially in women [11]. Additionally, the violent movement of the neck after surgery carries a risk of bleeding from the cauterized infrahyoid muscle, which leads to slow hemorrhage and hematoma formation [2]. Thus, reducing severe cough and emergence agitation can be helpful in decreasing postoperative bleeding. Therefore, we hypothesized that the effect of dexmedetomidine, which reduces cough and emergence agitation while the patient awakens, would decrease postoperative bleeding after thyroidectomy.

Various efforts have been made to reduce coughing during the time the patient awakens; such efforts include the administration of lidocaine either intravenously or topically, sub-hypnotic propofol, and remifentanil [12–14]. Dexmedetomidine has sedative and analgesic effects without significant respiratory depression and can be used during stressful procedures such as awake intubation [15]. Moreover, dexmedetomidine has recently gained attention as an adjuvant drug during emergence from anesthesia. In this study, we focused on the effects of dexmedetomidine. However, we omitted a loading dose as in previous studies of dexmedetomidine because of the possibility of sudden hemodynamic changes [4, 6].

Lee et al. reported that a single dose of dexmedetomidine (0.5 µg/kg for 10 min) with a low-dose remifentanil infusion (Ce of 1.0 ng/mL) at the end of thyroid surgery effectively suppresses cough during emergence and hemodynamic stability. However, only dexmedetomidine infusion at a rate of 0.4 µg/kg/h does not reduce the cough grade during emergence [6]. Unlike previous studies, in our study the administration of dexmedetomidine alone (0.6 µg/kg/h) without a loading dose during emergence from anesthesia resulted in a significant decrease in the cough reflex. The incidence of cough was significantly lower in group D (69.7 %) than in group S (82.9 %). In particular, the incidence of severe cough as grade 3 in group D decreased significantly compared to that in group S (4.3 % in group D and 11.5 % in group S). We considered gender as the cause of these differences. A previous study revealed that there are gender differences in the estimated EC50 of remifentanil for reducing cough during emergence, which was significantly lower in females than in males (1.30 ng/mL in females vs. 2.57 ng/mL in males) [16]. Unlike previous studies, all the subjects of our study were women. Therefore, in the present study, these gender differences were thought as being one of the factors that along with the use of only dexmedetomidine (0.6 µg/kg/h) had a sufficient effect on reducing the cough reflex. However, further research on gender-specific dexmedetomidine sensitivity is required.

It is well known that the sedative effect of dexmedetomidine is associated with a decreased incidence of emergence agitation. Dexmedetomidine administration (0.4 µg/kg/h during anesthesia) without a loading dose also provides smooth emergence after surgery and reduces emergence agitation [5, 6]. In the present study, we measured the RSS score to compare the emergence profiles, which revealed that dexmedetomidine resulted in calm awakening in the PACU (36.0 % in group D vs. 29.5 % in group S, *P* = 0.01). In particular, we classified RSS to compare the incidence of emergence agitation [9], and the results showed that dexmedetomidine decreased agitation (7.9 % in group D vs. 20.1 % in group S, *P* = 0.01) during the emergence period in the PACU. Despite the sedative effects, there were no significant differences in extubation time (*P* = 0.728) and recovery time (*P* = 0.604) and there was no event of desaturation after the administration of dexmedetomidine.

The unique result of this study was an assessment of postoperative bleeding. Our results showed a decrease in the amount of bleeding after thyroidectomy along with a reduction of the cough reflex and emergence agitation, although the was no significant difference in the duration of the drainage catheter placement. However, there is disagreement among studies regarding the effect of dexmedetomidine on perioperative bleeding. Dexmedetomidine decreases perioperative bleeding by maintaining a stable hemodynamic response during tympanoplasty or septoplasty, when administered as an adjuvant drug for the maintenance of anesthesia [17]. However, dexmedetomidine slightly increases perioperative bleeding after thyroidectomy in pediatric patients when administered before anesthesia induction (0.5 µg/kg) owing to its vasodilative effect as an α2 adrenergic agonist [18]. Moreover, the continuous infusion of dexmedetomidine attenuates the activation of coagulation in patients undergoing radical gastrectomy according to thromboelastography, by reducing the intraoperative stress response and an anti-inflammatory effect [19]. Nevertheless, in the current study, we administered a small dose of dexmedetomidine at the end of surgery when vascular ligation and bleeding control ended. A previous study also showed that a low dose of dexmedetomidine, similar to that used in our study, does not affect clotting profiles [18]. We assessed the amount of postoperative bleeding for three days and revealed a significant decrease in bleeding during emergence and while staying in the PACU (19.0 mL vs. 33.1 mL, *P* = 0.001), and the decrease of bleeding was confirmed until the second POD. Considering that hematoma usually occurs within 24 h after surgery [10], the difference in the amount of bleeding was statistically significant, although the absolute difference was relatively small (48.1 mL vs. 73.1 mL during the first 24 h). However, the size of the hematoma is not always proportional to the amount of bleeding [20], and hematoma formation in the deep layer of the neck, which compresses the airway, is lethal [21]. Therefore, efforts to reduce postoperative bleeding after thyroidectomy, such as reducing cough and decreasing emergence agitation, may be clinically necessary.

Additionally, dexmedetomidine decreased postoperative pain in the PACU in our study, although there were no significant differences in the NRS scores after the second POD. The analgesic effect of dexmedetomidine is well known, and intraoperative dexmedetomidine can effectively decrease postoperative pain [22]. Even low doses of dexmedetomidine (0.4 µg/kg/h infusion during laparoscopic surgery) result in a reduction in postoperative analgesic requirements [23]. According to a study by Yoo et al. [24], the intensity of postoperative pain after thyroidectomy is greatest at 30 min after surgery in the PACU and decreases by one-third after 24 h. As postoperative pain is identified as an independent risk factor for post-thyroidectomy hemorrhage [25], the analgesic effect of dexmedetomidine may also contribute to the reduction of postoperative bleeding. However, we only measured the intensity of postoperative pain on the first day in the PACU, which is considered a limitation of our study.

The administration of dexmedetomidine without a loading dose showed no significant differences in MBP between the two groups (*P* = 0.143), but the HR in our study was significantly lower before extubation compared to the control (*P* = 0.015). The infusion of a loading dose of dexmedetomidine can significantly increase blood pressure and decrease the heart rate [26]. We omitted the loading dose to prevent sudden hemodynamic fluctuations. Hemodynamic changes after the administration of dexmedetomidine vary according to individual variability and infusion methods. Lee et al. [4] showed no differences in MBP and HR compared to the control that received a small dose of dexmedetomidine without a loading dose. However, the infusion rate (0.6 µg/kg/h vs. 0.5 µg/kg/h) and duration of infusion (median 34 min vs. 10 min) of dexmedetomidine were higher and longer than those mentioned in the previous study. This difference in methods may have resulted in a decrease in heart rate without a difference in blood pressure.

This study has several limitations. First, only female patients were selected as the subjects of the study. This was because most of the patients with thyroid cancer were women in our hospital, as thyroid cancer is 2.9-times more common in women than in men [27]. Post-thyroidectomy cough is associated with females, as was mentioned above. Therefore, we restricted the study to women. However, drug sensitivity may be gender-specific, and the results of the current study are applicable only to women. Second, we did not evaluate postoperative nausea and vomiting (PONV). After thyroidectomy, PONV is a common complication and is associated with postoperative bleeding [2]. Adjuvant dexmedetomidine is effective in preventing PONV [28]. The dexmedetomidine used in our study may have reduced PONV and this could have been related to the outcome of our study, which was the reduction of postoperative bleeding. We only focused on the cough reflex and emergence agitation resulting from dexmedetomidine administration; therefore, further evaluation of PONV is required. Third, the optimal dosing method for dexmedetomidine should be evaluated. We used a low dose of dexmedetomidine for a relatively short time without a loading dose. Two previous studies and this study had different infusion rates and durations of dexmedetomidine [5, 6]. There are no guidelines yet for the appropriate infusion dose and rate of dexmedetomidine administration to reduce coughing or emergence agitation. Finally, the effects of dexmedetomidine may vary depending on the blood concentration of dexmedetomidine, and further research on the exact plasma concentration, infusion rate, and dosage is required.

## Conclusions

In conclusion, the administration of dexmedetomidine (0.6 µg/kg/h) without a loading dose during recovery from anesthesia decreased the incidence of severe cough and emergence agitation. These effects of dexmedetomidine can be helpful in reducing postoperative bleeding after thyroidectomy. However, further evaluation of the prevention of critical hemorrhage after thyroidectomy is required.

## Data Availability

The datasets analyzed during the current study are available from the corresponding author upon reasonable request.

## Abbreviations

ASA: American Society of Anesthesiologists
BIS: Bispectral index
Ce: Effect-site concentration
HR: Heart rate
MBP: Mean blood pressure
NRS: Numeric rating scale
PACU: Postanesthetic care unit
POD: Postoperative day
RSS: Ramsay Sedation Scale
TOF: Train-of-four
